# Kawasaki Disease and Respiratory Viruses: Ecological Spatiotemporal Analysis

**DOI:** 10.2196/49648

**Published:** 2024-07-25

**Authors:** Rana Sawires, Hazel J Clothier, David Burgner, Michael Collingwood Fahey, Jim Buttery

**Affiliations:** 1 Department of Paediatrics, Monash University, Wellington Road, Clayton, 380061 411717227, Australia; 2 Centre for Health Analytics, Murdoch Children’s Research Institute, Parkville, Australia; 3 Surveillance of Adverse Events Following Vaccination in the Community, Murdoch Children’s Research Institute, Parkville, Australia; 4 Melbourne School of Population and Global Health, University of Melbourne, Melbourne, Australia; 5 Department of Paediatrics, University of Melbourne, Melbourne, Australia; 6 Inflammatory Origins, Murdoch Children’s Research Institute, Parkville, Australia; 7 Department of Neurology, Monash Children’s Hospital, Clayton, Australia; 8 Neurogenetics Department, Monash Paediatrics, Monash University, Clayton, Australia

**Keywords:** Kawasaki disease, pediatric, infection, RSV, human metapneumovirus, respiratory virus, virology, community, viral infection, respiratory disease, respiratory diseases, children, epidemiology, respiratory syncytial virus

## Abstract

**Background:**

Kawasaki disease is an uncommon vasculitis affecting young children. Its etiology is not completely understood, although infections have been frequently postulated as the triggers. Respiratory viruses, specifically, have often been implicated as causative agents for Kawasaki disease presentations.

**Objective:**

We aimed to conduct an ecological spatiotemporal analysis to determine whether Kawasaki disease incidence was related to community respiratory virus circulation in a shared region and population, and to describe viral associations before and during the COVID-19 pandemic.

**Methods:**

We obtained independent statewide data sets of hospital admissions of Kawasaki disease and respiratory multiplex polymerase chain reaction tests performed at two large hospital networks in Victoria, Australia, from July 2011 to November 2021. We studied spatiotemporal relationships by negative binomial regression analysis of the monthly incidence of Kawasaki disease and the rate of positive respiratory polymerase chain reaction tests in different regions of Victoria. Peak viral seasons (95th percentile incidence) were compared to median viral circulation (50th percentile incidence) to calculate peak season increased rate ratios.

**Results:**

While no seasonal trend in Kawasaki disease incidence was identified throughout the study period, we found a 1.52 (99% CI 1.27‐1.82) and a 1.43 (99% CI 1.17‐1.73) increased rate ratio of Kawasaki disease presentations in association with human metapneumovirus and respiratory syncytial virus circulation, respectively, before the COVID-19 pandemic. No respiratory viral associations with Kawasaki disease were observed during the COVID-19 pandemic.

**Conclusions:**

Our large ecological analysis demonstrates novel spatiotemporal relationships between human metapneumovirus and respiratory syncytial virus circulation with Kawasaki disease. The disappearance of these associations in the COVID-19 pandemic may reflect the reduced circulation of non–SARS-CoV-2 viruses during this period, supporting the prepandemic associations identified in this study. The roles of human metapneumovirus and respiratory syncytial virus in Kawasaki disease etiology warrant further investigation.

## Introduction

Kawasaki disease is an uncommon acute systemic vasculitis, predominantly affecting children younger than 5 years [1]. Its defining features are fever lasting more than 5 days, bilateral nonpurulent conjunctivitis, changes to oropharyngeal mucous membranes, palmar and/or plantar erythema, polymorphous exanthema, and cervical lymphadenopathy [2]. It has been described with varying incidence worldwide [3]. In Australia, the annual Kawasaki disease incidence in children ages 0‐4 years is 14.31/100,000, as measured by the rate of intravenous immunoglobulin treatment [4]. The etiology is poorly understood, with many putative causes proposed, investigated, and excluded [3,5]. However, one or more preceding infective causes are widely suggested as the trigger for the disease and fit with epidemiology [1,3,6,7]. Local outbreaks in two cities in the United States in 1979 and 1980 showed that the only difference between patients and controls was a history of respiratory illness, which may imply concurrent circulation of a viral infectious trigger [8]. Since then, seasonal patterns of Kawasaki disease have been observed in Japan, Korea, and France, with annual peaks occurring in winter and an additional peak in summer in Japan [9-11]. In Victoria, Australia, Kawasaki disease historically peaks in late winter and early spring, which has also been described in studies from the northern hemisphere [4,9]. The co-occurrence of respiratory viral infections in conjunction with Kawasaki disease has been variously reported [12]. A prospective study in Taiwan found that, in addition to a greater prevalence of respiratory symptoms among children with Kawasaki disease, there was a higher rate of polymerase chain reaction (PCR) viral detection in patients with Kawasaki disease compared to controls, including enteroviruses (16.8% vs 4.4%), rhinoviruses (26.5% vs 9.7%), adenoviruses (8% vs 1.8%) and non–SARS-CoV-2 coronaviruses (7.1% vs 0.9%) [13]. Observational studies have reported an association between Kawasaki disease and adenovirus [14], human rhinovirus [15], and non–SARS-CoV-2 coronavirus [16]. An ecologic study in South Korea of over 53,000 Kawasaki disease cases recently identified temporal correlations between rhinovirus, respiratory syncytial virus (RSV), and varicella peaks 1‐3 months preceding Kawasaki disease outbreaks [17]. Reductions in Kawasaki disease presentations in the United States during the COVID-19 pandemic further support the role of respiratory viruses in this disease [18], given that its decline was coincident with reduced respiratory virus circulation throughout the pandemic. Conversely, the appearance of pediatric inflammatory multisystem syndrome temporally associated with SARS-CoV-2 (PIMS-TS) throughout the COVID-19 pandemic has compounded the complexity of a Kawasaki disease diagnosis, as PIMS-TS clinically resembles Kawasaki disease [19-21].

Given the variability of viral associations with Kawasaki disease that have been identified to date and the methodological limitations of some previous studies, we aimed to investigate spatiotemporal associations of Kawasaki disease incidence with respiratory virus circulation in Victoria, Australia over 10 years.

## Methods

### Study Design and Cohort

We conducted a retrospective ecologic cohort analysis from July 2011 through November 2021 using independent, unlinked data sets from Victorian hospitals to define associations between Kawasaki disease and respiratory virus circulation.

Kawasaki disease admissions data were obtained from the Victorian Agency for Health Information (VAHI), a state government organization that collates, analyzes, and shares health data from public hospitals across the Australian state of Victoria (population 6.5 million) [22]. VAHI collates data from the Victorian Admitted Episodes Dataset (VAED), which contains data from Victorian public hospital admissions. Data are recorded according to the *International Statistical Classification of Diseases and Related Health Problems, Tenth Revision, Australian Modification* (*ICD-10-AM*). *ICD-10-AM* code M303 was used to extract any presentation for which the primary diagnosis was Kawasaki disease. We extracted fields related to date of presentation; patient area of residence, aggregated to Statistical Area Level 3 (SA3); and duration of stay. SA3s are regional breakdowns of Australia, with populations ranging from 30,000 to 130,000 people [23]. Kawasaki disease presentations in children ages 0‐9 years were included in this analysis. VAHI ethics restrictions preclude the use of narrower age bands (eg, 0‐4 and 5‐9 years), as this may breach patient confidentiality due to the low incidence of Kawasaki disease once restricted to month and SA3. We were unable to account for interhospital transfers or repeat presentations for the same episode as individual patients cannot be identified from these data. Kawasaki disease presentations were aggregated by month of presentation and patient area of residence (SA3).

We obtained PCR results for common respiratory viruses from two large tertiary hospitals in Victoria, Australia. Monash Health (MH), based in southeast Melbourne, is the largest health network in Victoria, with almost 40,000 annual pediatric admissions across three emergency departments [24]. The Royal Children’s Hospital Melbourne (RCH) is the largest children’s hospital in Australia, with care extending to children from Tasmania, New South Wales, and other Australian states. RCH has almost 55,000 admissions annually, with various other nonadmission services [25]. From these two tertiary hospitals, respiratory multiplex PCR assays reported results for the following viruses: adenovirus; influenza A and B; parainfluenza 1, 2, 3, and 4; SARS-CoV-2 (from March 2020 onward); human metapneumovirus (HMPV), RSV, parechovirus, and picornavirus. Data for all reported viruses were included in this study. Data included date of test, patient area of residence aggregated to SA3, and PCR test result. Respiratory PCR data were aggregated by month of presentation and patient area of residence (SA3).

Our inclusion criteria were all respiratory multiplex PCR assays performed at MH and RCH for patients of any age, both children and adults, with a Victorian residential SA3. All Kawasaki disease presentations recorded for children younger than 10 years were included from the VAED (ie, all Victorian hospitals) between July 1, 2011, and November 30, 2021. Individual patient records were not available for analysis to ensure patient deidentification. However, a previous study of Kawasaki disease epidemiology in Australia using linked total population data has been verified against patient records [26].

### Statistical Analysis

The coding program R (version 4.0.2; The R Foundation for Statistical Computing) [27] was applied through RStudio (version 1.2.5; Posit PBC) [28] for statistical analysis of temporal data. To account for the significant increase in total PCR tests performed each year at RCH and MH, viral incidence data were converted into a monthly rate per number of positive PCR tests.

We modified a statistical approach developed under the Snotwatch framework, which has been previously used to address viral associations with febrile seizures and chilblain presentations [29,30]. We created four models for our data sets, applying a negative binomial regression technique. Two models assessed the spatiotemporal trends of Kawasaki disease from 2011 to 2019 (pre–COVID-19 pandemic), and two assessed the spatiotemporal trends of Kawasaki disease during the COVID-19 pandemic (2020‐2021). For each period, we created a model that used the month of the year as an independent predictor of Kawasaki disease, allowing us to determine the trend in Kawasaki disease presentations throughout the year. All increased rate ratios in the month-of-the-year model were relative to the average number of Kawasaki disease presentations in April, which was selected as the mid-autumn reference month to allow comparison with seasonal variations in winter and summer.

We also created a multivariate model that used all positive respiratory virus PCR tests as separate predictors of Kawasaki disease. This model enabled us to ascertain which viruses were related to Kawasaki disease presentations in space and time over the study period. For each virus, the number of positive tests in any given month was divided by the total number of PCR tests performed during that month.

We compared the association of peak virus circulation on Kawasaki disease presentations with the median expected virus circulation. Specifically, we compared the risk of Kawasaki disease when viruses were present at or above the 95th percentile of their maximum rate to the 50th percentile of their maximum rate.

### Ethics Approval

An informed consent waiver and ethical approval through our primary human research ethics committee was obtained from MH on July 24, 2019 (NMA/ERM reference RES-19‐0000333L-53611).

## Results

### Overview

**Figure 1.**
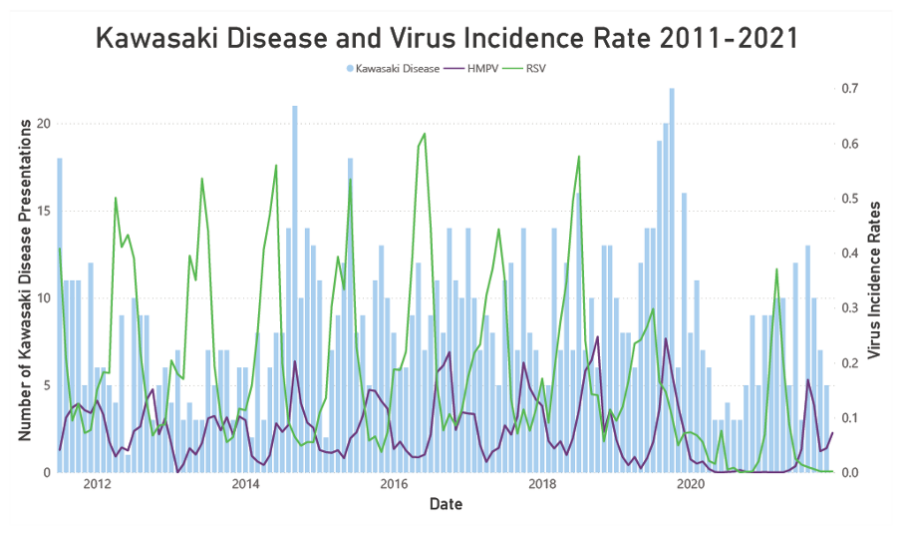
Number of Kawasaki disease presentations and HMPV and RSV monthly incidences in Victoria, Australia, from 2011 to 2021. HMPV: human metapneumovirus; RSV: respiratory syncytial virus.

From July 2011 to November 2021, there were 1081 presentations of Kawasaki disease in children younger than 10 years, of which 873 (80.8%) resided in metropolitan Melbourne. Kawasaki disease incidence in the 2 years 2020 and 2021 represented 160 (14.8%) of all presentations over the total study period. We obtained 153,153 positive respiratory multiplex PCR tests from MH and RCH conducted between July 2011 and November 2021, of which 14,032 (9.4%) included SARS-CoV-2 detections from January 2020 to November 2021. Kawasaki disease’s average monthly incidence decreased by 39% in 2020 and 7.7% in 2021 compared to the prepandemic monthly average. Figure 1 demonstrates the temporal incidence of Kawasaki disease and the HMPV and RSV incidence rates throughout the study period.

### Temporal Associations

In our analyses of temporal trends of Kawasaki disease, we found no associations between the month of year and Kawasaki disease incidence both before and during the COVID-19 pandemic years.

### Spatiotemporal Associations Between Viral and Kawasaki Disease Incidence

Negative binomial regression analysis demonstrated two viral associations with presentations of Kawasaki disease in Victoria before the COVID-19 pandemic. We found a 1.52 (99% CI 1.27‐1.82) increased risk of Kawasaki disease presentations in peak seasons of HMPV and a 1.43 increased rate ratio (99% CI 1.17‐1.73) in peak RSV seasons. During the pandemic (2020 onward), no associations between viral incidence and statewide Kawasaki disease presentations were observed (Table 1).

**Table 1.** Spatiotemporal viral associations with Kawasaki disease in Victoria, Australia, from 2011 to 2021.

Virus	2011‐2019 IRR^a^ (99% CI)	*P* value	2020‐2021 IRR (99% CI)	*P* value
Adenovirus	1.06 (0.87‐1.30)	.45	0.96 (0.45‐2.07)	.89
COVID-19	—^b^	—	1.00 (1.00‐1.00)	.15
Human metapneumovirus	1.52 (1.27‐1.82)	<.001	0.99 (0.92‐1.07)	.78
Influenza A	1.12 (0.99‐1.26)	.02	1.03 (0.91‐1.18)	.49
Influenza B	1.02 (0.91‐1.13)	.71	1.00 (1.00‐1.00)	.37
Parechovirus	1.00 (1.00‐1.00)	.20	1.00 (1.00‐1.00)	.55
Picornavirus	0.91 (0.70‐1.18)	.34	1.21 (0.70‐2.10)	.38
Parainfluenza	1.01 (0.93‐1.10)	.76	1.01 (0.78‐1.32)	.92
Respiratory syncytial virus	1.43 (1.17‐1.73)	<.001	1.16 (1.00‐1.35)	.01

a IRR: increased rate ratio.

b Not applicable.

## Discussion

This is a large-scale population-based ecological spatiotemporal analysis of Kawasaki disease in conjunction with respiratory viral circulation over 10 years. We found a novel association between Kawasaki disease and HMPV and (separately) with RSV. The RSV association is similar to the findings of Kang et al [17] using a different temporal ecologic analysis, although our spatiotemporal association was contemporaneous, while their temporal association supported a 1‐ to 3-month lag between outbreaks and Kawasaki disease peaks. Despite being associated with HMPV and RSV, viruses that commonly peak in the winter of each year, there were no independent associations between Kawasaki disease incidence and seasons. This adds credence to the association between Kawasaki disease and these two viruses, suggesting a closely related temporal co-occurrence that cannot be generalized to the month of incidence [9]. The spatial distribution of the data sets is as expected and suggests the relatively equal likelihood of Kawasaki disease presentations across the state of Victoria.

In contrast with our study, Kang et al [17] also identified associations with varicella and rhinovirus. Our laboratory assays identified picornavirus circulation, which includes both rhinovirus and enteroviruses—two viruses that have contrasting seasonal trends and consequently may not have been able to be associated with Kawasaki disease by our model, potentially accounting for the discrepant findings [31]. Varicella was not included in our analysis, and the childhood varicella vaccine has been implemented in Australia since 2005, with rare outbreaks [32].

We did not observe any viral associations with Kawasaki disease incidence throughout the pandemic, which may, in part, have reflected the lockdown restrictions in Victoria from 2020 to 2021 that altered viral circulation in the community [33] and quantifiably reduced infection-related hospitalization in children by 65% [34-36]. The reduction in monthly presentations of Kawasaki disease throughout the pandemic years has been reported internationally, supporting a putative role for transmissible viruses in Kawasaki disease etiology [13,37-41] but not supporting the suggestion that SARS-CoV-2 infections may cause Kawasaki disease [42]. Nevertheless, the fact that there were ongoing presentations of Kawasaki disease despite the limited viral transmission in this period also suggests that Kawasaki disease etiology is more complex [43]. Pitzer et al [7] have suggested that Kawasaki disease onset may be triggered by two concurrent infections; an acute infectious agent (such as a respiratory virus) and an infection with a longer duration (such as a superantigen elaborating colonizing bacteria). A plausible alternative interpretation is that one or more infectious agents that are not included in our multiplex PCR assay and for which transmission was not substantially reduced by the lockdowns are also responsible for Kawasaki disease cases [44,45]. The lack of association between SARS-CoV-2 and Kawasaki disease in our study suggests that Kawasaki disease is not related to PIMS-TS as was initially suggested [19-21]. Our ecologic association findings may inform future investigations of the possible mechanistic roles of respiratory viruses in Kawasaki disease etiology.

We acknowledge some limitations in this study. An important limitation of our study was the skewed geographical distribution of the respiratory PCR test data. These two hospitals had broad coverage of respiratory PCR test data, with virus assay results demonstrated for patients residing in all SA3s in Victoria. However, virus circulation data predominated in the eastern, inner, and inner western suburbs of Melbourne, where these hospital networks are located. MH also provides pathology services for eastern and southern Victoria, including respiratory PCR assays. As such, this introduced potential bias into the analysis. The associations identified in this study may be an over- or underrepresentation of the actual viral associations with Kawasaki disease due to insufficient viral data representation in some parts of the state. An additional limitation was that Kawasaki disease presentation data were obtained from statewide administrative hospital admissions records. Admissions data for Kawasaki disease may overestimate true disease episodes, as was demonstrated in a comparison of emergency department and hospital admission diagnoses in 2015, with clinicians possibly overdiagnosing Kawasaki disease to avoid missing this important diagnosis [46,47]. However, hospitalization data and intravenous immunoglobulin use for Kawasaki disease correlate highly in Australia [4].

An important consideration is that this study used viral infections in Victoria as predictors of Kawasaki disease, irrespective of the host age. We chose this approach because all-population viral infections were used as a surrogate of potential exposure to all circulating microorganisms, in contrast to true infection. We also recognized that restricting the age group of patients with positive respiratory PCR tests would give us a representation of viral infections in children severe enough to lead to hospitalization rather than any virus exposure or mild infection. Notably, PCR data obtained from RCH would represent viral infections in children predominantly (with the occasional adult test conducted on site for parents or guardians). We recognize that this would skew test results toward respiratory viruses that are more common in children due to a sampling bias. Consequently, PCR data used in this analysis may not accurately represent potential exposure to all circulating microorganisms. However, the larger sample size in itself may improve the accuracy of the results, and this has been demonstrated in reviews of nonhealth data [48]. Additionally, data completeness is only one of several dimensions of data quality, and other features including amount of data, consistency, geographic coverage, usability, and maintainability are other important features that these PCR data possess [49].

We have identified potential viral triggers of Kawasaki disease and demonstrated the application of our novel statistical approach. While ecologic studies can only inform association rather than causation, these findings may be used to inform alternate etiological methodologies. We have therefore demonstrated the utility of the Snotwatch ecological data platform to investigate an uncommon disease and to identify novel associations with common viral pathogens. If these findings are validated, with the rapid clinical development of RSV vaccines and the recent revolution in vaccine technologies, we look forward to a time when Kawasaki disease may become (at least partially) a vaccine-preventable disease.

This spatiotemporal analysis of Kawasaki disease identified two potential associations between Kawasaki disease and HMPV and RSV before the COVID-19 pandemic in Victoria, Australia. We used a novel statistical technique to investigate an uncommon disorder at a population level. The additional granularity of the spatial component of our analysis adds accuracy to our findings by exploring associations within each region’s cohort, while the large sample size adds power. Future investigation of respiratory virus associations with Kawasaki disease may confirm that RSV and HMPV have etiological roles in Kawasaki disease and are important targets for intervention.

## Abbreviations

HMPV: human metapneumovirus
*ICD-10-AM*: *International Statistical Classification of Diseases and Related Health Problems, Tenth Revision, Australian Modification*
MH: Monash Health
PCR: polymerase chain reaction
PIMS-TS: pediatric inflammatory multisystem syndrome temporally associated with SARS-CoV-2
RCH: Royal Children’s Hospital Melbourne
RSV: respiratory syncytial virus
SA3: Statistical Area Level 3
VAED: Victorian Admitted Episodes Dataset
VAHI: Victorian Agency for Health Information
